# Rust Distribution of Non-Uniform Steel Corrosion Induced by Impressed Current Method

**DOI:** 10.3390/ma15124276

**Published:** 2022-06-16

**Authors:** Qiang Li, Zhiji Gao, Tao Yang, Zheng Dong, Zhilu Jiang, Qi He, Chuanqing Fu

**Affiliations:** 1 College of Civil Engineering and Architecture, Zhejiang University of Water Resources and Electric Power, Hangzhou 310018, China; liq@zjweu.edu.cn; 2 College of Civil Engineering, Zhejiang University of Technology, Hangzhou 310014, China; gzhiji2022@163.com (Z.G.); 2112106093@zjut.edu.cn (T.Y.); zdong@zjut.edu.cn (Z.D.); chqfu@zju.edu.cn (C.F.); 3 College of Civil Engineering and Architecture, Zhejiang Tongji Vocational College of Science and Technology, Hangzhou 311231, China; heqiqihe@yeah.net

**Keywords:** non-uniform corrosion, thickness distribution of rust layer, cracking pattern, finite element analysis

## Abstract

The non-uniform corrosion of steel bars is the main factor affecting the durability of concrete. The cracking pattern of concrete due to corrosion is closely related to the distribution of the corrosion products. Research on the thickness distribution of the rust layer and the cracking pattern of concrete under different influencing factors is of great significance in the prediction of the service life of existing reinforced concrete structures and the avoidance of the premature cracking of the reinforced concrete structures to be built. This paper studies the thickness distribution of the rust layer on the surface of single and multiple corroded reinforcements under non-uniform corrosion. The electrochemical analysis of the electrified corrosion process was carried out by using the finite element analysis software, and the distribution of the current density was obtained. The effects of geometric parameters, steel bar position, and steel bar spacing and shape on the corrosion expansion cracking pattern were studied. The results indicated that as the position of the steel bar differed, the crack pattern of the concrete changed, depending on the number of corrosion peaks (i.e., the maximum thickness of the rust layer). In terms of the corner-located steel, the number of corrosion peaks varied in the cases of different geometrical parameters (i.e., the diameter of the steel bar and the distance between the steel bars and the stainless steel wire). Nevertheless, the critical corrosion degrees of the side-located and corner-located steel bars, with respect to the cracking of the outer concrete surface, were basically the same. Additionally, the ribbed steel bar presented a lower critical corrosion degree than that of the plain steel bar, while little influence was exhibited with the varying angles of the rib.

## 1. Introduction

In order to maintain the long-term performance of a reinforced concrete structure, the detection and monitoring of the defects and degradation have attracted increasing attention. In terms of the durability of a reinforced concrete structure, steel corrosion is the main reason for the structural deterioration [1,2,3]. The corrosion of steel bars in concrete in the natural environment shows non-uniform corrosion, and the cracking pattern of concrete under non-uniform corrosion is very different from that of the uniform corrosion. The corrosion morphology has an important effect on the mechanical properties of the reinforcing steel bars [4]. Therefore, in considering the characteristics of the non-uniform corrosion of steel bars, it is of great significance to study the critical corrosion rate, the cracking time, and the key characteristic parameters of the concrete cracking pattern for the durability design, the service life prediction, and the maintenance and reinforcement of reinforced concrete structures.

The current experimental research methods on reinforcement corrosion include the natural environment corrosion test method [5], the artificial simulated environment accelerated corrosion test method, and the electrochemical accelerated corrosion test method [6]. The corrosion expansion crack pattern of reinforced concrete obtained by the natural environment corrosion test method is the closest to the actual service condition, but the test period is long. Fu et al. [5] exposed the specimens with transverse initial cracks to the natural environment for four years and found that the existence of transverse cracks exacerbated the corrosion of the tensile steel bars. Poupard et al. [7] studied the corrosion degree of steel bars in various positions by visual measurement and chloride ion content in reinforced concrete beams exposed to the natural tidal environment for 40 years. It was found that the corrosion degree of the steel bars in the tensile zone of the beam was higher than that in the compression zone. The reason was that microcracks were produced in the tensile zone, which increased the permeability of the chloride ion and oxygen. Comparatively, the accelerated corrosion test in an artificial environment has a shorter test period. The results of Ye et al. [8] showed that the corrosion characteristics obtained by the artificial environment accelerated corrosion test method were closer to the natural conditions. With the same crack width, the corrosion degree of the steel bars obtained in the artificial environment is lower than that of the electrochemical accelerated corrosion test. The electrochemical accelerated corrosion test method is widely used in the research of steel corrosion due to its short testing time, convenient instruments, and operation with high repeatability. However, the correlation between the results obtained by the electrochemical accelerated corrosion test method and the natural conditions is not clear. In the initial version of the electrochemical accelerated corrosion test, only uniform corrosion was obtained. The distribution of the steel rust layer under uniform corrosion is quite different from that under natural corrosion. In order to obtain the results of the non-uniform corrosion of steel bars, the electrochemical test methods commonly used are the full immersion method [9], the half immersion method [10,11], and the built-in auxiliary electrode method [12,13]. The morphology of the corrosion pits can be measured by 3D scanning methods [14]. Compared with the experimental study on the steel rust layer and the concrete cracking mode, the finite element analysis method can greatly reduce the test workload, with sufficient precision and high repeatability. In the finite element simulation on steel corrosion by researchers, the steel corrosion is usually regarded as uniform corrosion [15]; that is, assuming that the thickness distribution of the rust layer in the circumferential direction of steel is consistent, a uniform rust expansion force is generated, and the cracking pattern of concrete is studied on this basis. Recently, the expansion of non-uniform steel corrosion was modeled based on a chemical–mechanical computational framework [16].

The widely used electrical accelerated corrosion test method is not able to form non-uniform corrosion of steel bars, and the rust layer morphology is not the same as that under the natural environment. Additionally, the influence of the various test parameters based on this test method on the distribution of the steel rust layer and the concrete cracking pattern is not fully understood. To this end, the purpose of this study is to explore the effect of the test parameters on the rust distribution and the cracking pattern of concrete based on a simulation method. In this paper, an electrochemical accelerated corrosion test method is simulated for the non-uniform corrosion of steel bars. Combined with the finite element analysis method, the corrosion process of steel bars and the cracking process of concrete in the non-uniform corrosion method are analyzed.

## 2. Non-Uniform Accelerated Corrosion Method

The accelerated corrosion methods for simulating the non-uniform corrosion of steel bars include the full immersion method, the half immersion method, and the built-in auxiliary electrode method. Due to the distance between the two electrodes for the full immersion and the half immersion methods, the non-uniformity of steel corrosion is not obvious, and the corrosion efficiency is low. Compared with the former two methods, the distance between the two electrodes of the built-in auxiliary electrode method is closer and the resistance is smaller; so, the non-uniformity of the steel corrosion is the strongest and the corrosion efficiency is the highest.

**Figure 1:**
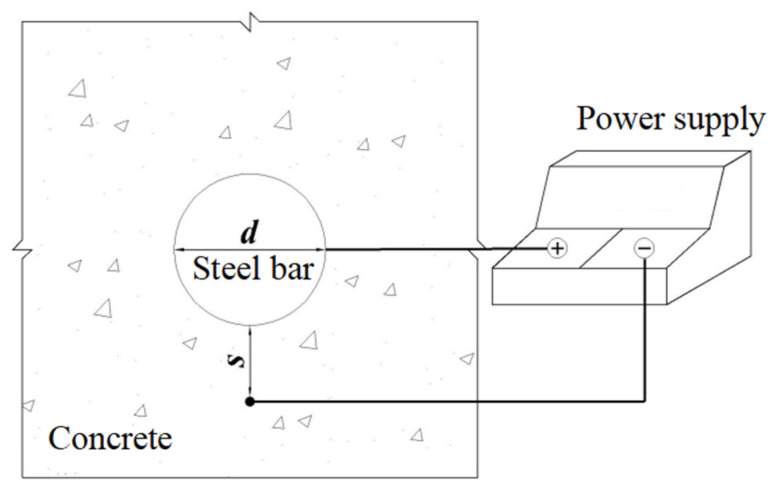
Schematic diagram of the new non-uniform corrosion method.

In order to further improve the built-in auxiliary electrode method, stainless steel wire with a small section area, instead of a stainless steel rod, is placed near the reinforcement in the concrete cover as the auxiliary electrode. The steel bar is used as the positive electrode of the anode and the stainless steel wire is used as the negative electrode connected to a DC power supply. Because the electron transport rate is much faster than the cathode reaction rate, a large number of electrons will gather around the cathode, resulting in an electron-rich phenomenon. The cathode area is much smaller than the anode area, and the distance between the two electrodes is close. Due to the effect of the Coulomb force, the closer the anode is to the cathode, the greater the Coulomb force is, and the electron is preferentially lost due to the repulsion force. The metal cations preferentially enter the solution due to the force; so, the steel bars show non-uniform corrosion. The schematic diagram of the non-uniform corrosion method is shown in Figure 1. The diameter of the stainless steel wire is 0.8 mm; the diameter of the steel bar is designated as *d*; and the net distance between the steel bar and the stainless steel wire is designated as *s*. The parameters *s* and *d* are important because they can affect the thickness distribution of the rust layer on the surface of the steel bar.

## 3. Corrosion Thickness Distribution under Non-Uniform Accelerated Corrosion

### 3.1. Basic Principles of Modeling

The accumulation of corrosion products in the process of electric corrosion conforms to Faraday’s law [17], as shown in Equation (1). The distribution function of the current density in the circumferential direction of reinforcement is defined as *i*(*θ*), and the distribution function of the rust thickness is defined as *T_r_*(*θ*). The geometric relationship between the current density distribution and the current is expressed by Equation (2). The geometric relationship between the thickness distribution of the rust layer and the volume of the rust product is given by Equation (3). The relationship between the current density and the thickness distribution of the rust layer can be obtained by combining Equations (1)–(3), resulting in Equation (4). Equation (4) indicates that in the accelerated corrosion test, the distribution of the surface current density determines the distribution of the rust layer thickness. Consequently, the key to understanding the distribution of the rust layer thickness in the electric corrosion process is to explore the distribution of the surface current density.

(1) $$V=\frac{n\cdot M_{\mathrm{Fe}}\cdot I\cdot t}{Z_{\mathrm{Fe}}\cdot F\cdot \gamma_{{}_{S}}}$$

(2) $$I=\frac{d}{2}\cdot L\cdot \int_{-\pi }^{\pi }i\left(\theta ,d\right)d\theta$$

(3) $$V=\frac{d}{2}\cdot L\cdot \int_{-\pi }^{\pi }T_{cl}\left(\theta \right)d\theta$$

(4) $$T_{r}\left(\theta \right)=\frac{n\cdot M_{\mathrm{Fe}}\cdot t}{Z_{Fe}\cdot F\cdot \gamma_{s}}\cdot i\left(\theta \right)=\frac{n\cdot d\cdot \rho }{4i_{\mathrm{ave}}}\cdot i\left(\theta \right)$$

where *V* is volume of the corrosion products (m^3^); *n* is the expansion coefficient of the rust products, which is related to the composition of the corrosion products, usually given as 2–6 [18,19]; *M*_Fe_ is the molar mass of iron (*M*_F_ = 56 g/mol); *I* is the constant current (A); *t* is the test time (s); *Z*_Fe_ is the combined valence of iron (*Z*_Fe_ = 2); *F* is the Faraday constant (*F* = 96,485 C/mol); *γ_s_* is the steel density (*γ_s_* = 7.85 × 10^6^ g/m^3^); *d* is the initial diameter of the reinforcement (m); *l* is the length of the electrified area of the steel bar (m); *ρ* is the corrosion rate of the steel bar; and *i*_ave_ is the average current density on the steel surface (A/m^2^).

In the process of electric corrosion, the two electrodes will change from equilibrium potential to polarization potential, which is called polarization. The degree of polarization of an overpotential reaction is the difference between the polarization potential and the equilibrium potential:

(5) $$\eta_{a/c}=E_{a/c}-E_{a/c}^{0}$$

where *η_a/c_* is the overpotential; *E_a/c_* is the polarization potential; and *E*^0^*_a/c_* is the equilibrium potential. The subscript *a* is the anode and *c* is the cathode. The electrode polarization type of the test method in this paper is electrochemical polarization [20], and the relationship between the current density and overpotential satisfies the Butler–Volmer equation [21]:

(6) $$i_{a}=i_{a}^{0}\cdot \left[\exp(\frac{\gamma mF}{RT}\cdot \eta_{a})-\exp(\frac{\alpha mF}{RT}\cdot \eta_{a})\right]$$

(7) $$i_{c}=i_{c}^{0}\cdot \left[\exp(\frac{\alpha mF}{RT}\cdot \eta_{c})-\exp(\frac{\gamma mF}{RT}\cdot \eta_{c})\right]$$

where *i_a_* and *i_c_* are the corrosion current densities of the anode and cathode, respectively; *i*^0^*_a_* and *i*^0^*_c_* are the exchange current densities of the anode and cathode, respectively; *α* and *γ* are the charge transfer coefficients of the anode and cathode, respectively; *m* is the combined price; *F* is the Faraday constant; *R* is a gas constant; and *T* is the thermodynamic temperature.

The corrosion rate is often controlled by the average current density of the steel bars in the test process; so, boundary conditions should be added to calculate the current density:

(8) $$\frac{d_{a}}{2}\cdot \int_{-\pi }^{\pi }i_{a}d\theta =\frac{d_{c}}{2}\cdot \int_{-\pi }^{\pi }i_{c}d\theta =\pi d_{a}\cdot i_{\mathrm{ave}}$$

where *d_a_* and *d_c_* are the diameters of the anode and cathode, respectively, and *i*_ave_ is the average current.

**Figure 2:**
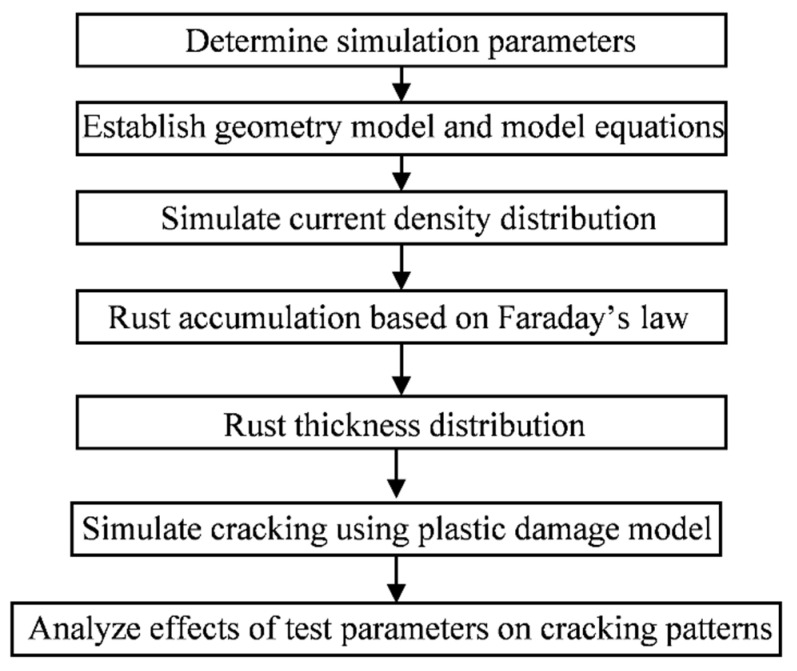
Flowchart for the modeling work.

In order to explore the influence of the diameter of the steel bar and the net distance of the two electrodes on the current density distribution of the steel bar surface under the non-uniform corrosion method, finite element simulation is used to analyze the process of the electric corrosion of the steel bar by COMSOL Multiphysics software. The flowchart of the modeling work is shown in Figure 2. In Equations (5)–(8), parameters *α* and *β*, *i*^0^*_a_* and *i*^0^*_c_* are related to the chemical reaction conditions, which can be determined by experimental methods, and *E*^0^*_a/c_* can be calculated by the Nernst equation. The corrosion current density of the two electrodes can be determined by the polarization potential of the two electrodes. Based on the above experimental environment and electrochemical analysis, the electrochemical parameters used in the model are shown in Table 1. The specimen with concrete C30 (the standard value of concrete strength was 30 MPa) was studied. The specimen was immersed in a 3.5% sodium chloride solution for three days, and a sponge coating with a sodium chloride solution was used to cover the specimen during the electrification process to ensure that the specimen was filled with solution. According to the existing experimental studies, when the current density is less than or equal to 3 A/m^2^, the test results of the rust expansion cracking of the reinforced concrete are closer to those under a natural exposure environment [13]; so, the average current density of the reinforcement is 3 A/m^2^.

### 3.2. Current Density Distribution in Circumferential Direction of Steel Bar

**Figure 3:**
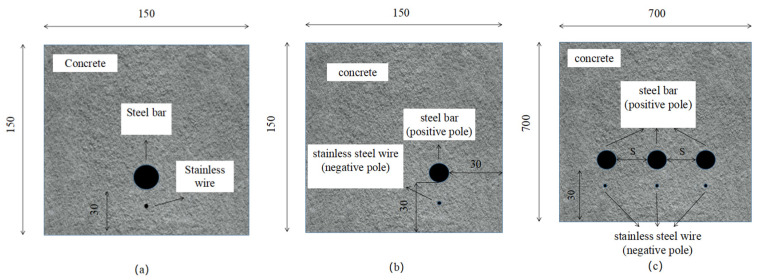
Geometric model of finite element simulation of electric corrosion: (**a**) single steel bar in unilateral middle; (**b**) unilateral corner single reinforcement; (**c**) single row of multiple reinforcements.

The electric corrosion process of concrete specimens with a single steel bar in the middle of one side (Figure 3a) or at a corner (Figure 3b) and a single row of multiple steel bars (Figure 3c) is simulated. The dimensional size of the concrete specimen is shown in Figure 2. The thickness of the specimen is 75 mm, and the thickness of the concrete cover is 30 mm. The steel bar and the stainless steel wire are the anode and cathode, respectively. The specimens in Figure 3a,b are used to explore the influence of the diameter (*d*) of the steel bar and the net distance (*s*) between the two electrodes on the current density distribution on the steel surface. The diameters of the steel bars were 8, 12, 16, 24, and 32 mm, and the net distances between the two electrodes were 4, 8, 12, 16, and 20 mm, respectively. For the arrangement of the corner reinforcement, the corrosion medium of the thickness of the protective layers on both sides will reach the surface of the reinforcement at the same time in the process for natural corrosion, and the surface of the reinforcement on one side of the two protective layers will begin to rust at the same time. Therefore, in the simulation process, the stainless steel wires need to be built in the protective layers on both sides, as shown in Figure 3b. When exploring the current density distribution function in the circumferential direction of multiple rebars, the influence of the adjacent rebars on the current density distribution of the tested rebars is mainly explored. The main parameters are rebar diameter *d* and net distance *S* between the rebars. In Figure 3c, the middle steel bar is the tested steel bar, and the position of the two sides of the steel bar is changed to vary *S*. The diameters of the steel bars are set as 8, 12, 16, 24, and 32 mm, respectively. The net distance of the reinforcement is expressed in multiples of d, i.e., *d*, 2*d*, 4*d*, 6*d*, and 8*d*.

#### 3.2.1. Unilateral Positioning Steel Bar

**Figure 4:**
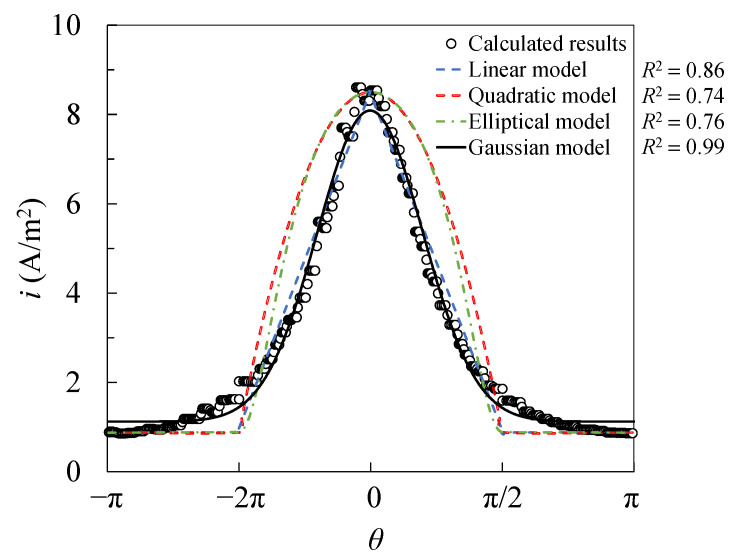
Regression analysis of the proposed model with calculation results.

The calculated results of the corrosion current density distribution of the steel bars in the middle of the single side are shown in Figure 4. The results of the circumferential current density are fitted by different formulas, as shown in Figure 4. The comparison shows that the calculation results can be well fitted by the Gaussian function:

(9) $$i\left(\theta \right)=y+T_{m}\cdot e^{{-\left(\frac{\theta }{\sqrt{2}\cdot c}\right)}^{2}}$$

where *y* defines the minimum current density; *T_m_* represents the inhomogeneity of the current density, which is related to the difference between the maximum and minimum current densities; and *c* reflects the width of the Gaussian curve, which can be described as the distribution of the corrosion current density.

**Figure 5:**
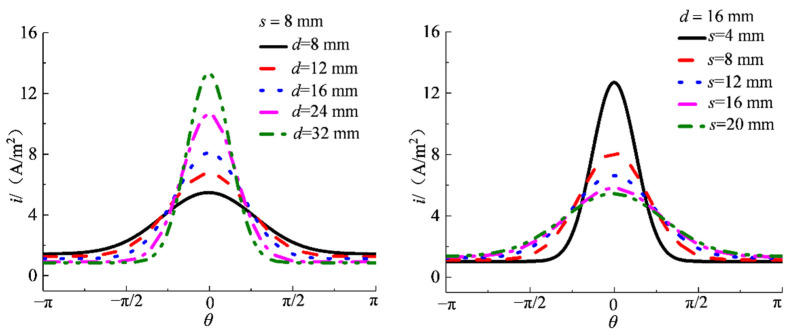
Current density distribution with *d* and *s*.

The typical results of the current density around the side bars with different *d* and *s* are shown in Figure 5. With the increase in the steel bar diameter, the maximum current density increases, while the minimum current density decreases, indicating that the uneven distribution of the current density increases. As the distance between the steel bar and the stainless steel wire increases, the trend is the opposite. Table 2 lists the fitting values of the parameters in the equation under different *d* and *s*. According to the results in Table 2, the relationships *y*(*d*,*s*), *T_m_*(*d*,*s*), and *c*(*d*,*s*) between *y*, *T_m_*, *c* and *d* and *s* are obtained, as shown in Equations (10)–(12), where the unit of *s* and *d* is m. Finally, the current density distribution model of the single middle steel bar under the condition of electric corrosion is Equation (13).

(10) $$y\left(d,s\right)=1.53+53.28s-57.45d+1088.62d^{2}-1147.52s\cdot d$$

(11) $$T_{m}\left(d,s\right)=6.85-1139.08s+621.81d+46107.14s^{2}-25126.67s\cdot d$$

(12) $$c\left(d,s\right)=0.67+64.98s-37.37d-1470.53s^{2}+495.92d^{2}+4.09s\cdot d$$

(13) $$i\left(\theta ,d,s\right)=y\left(d,s\right)+T_{m}\left(d,s\right)\cdot e^{-\left(\frac{\theta }{\sqrt{2}\cdot c\left(d,s\right)}\right)}{}^{2}$$

#### 3.2.2. Single Steel Bar at Corner

**Figure 6:**
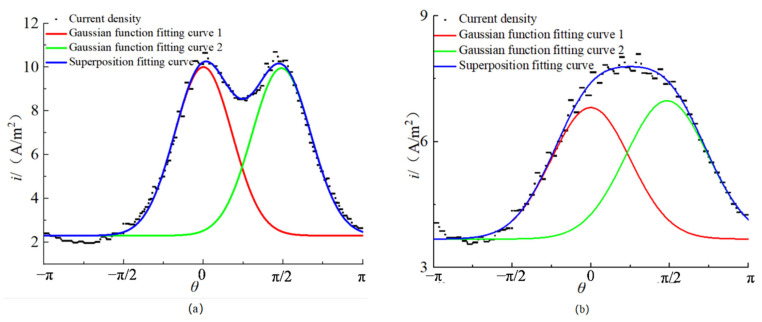
The surface current density distribution of single corner reinforcement: (**a**) double peaks; (**b**) single peak.

For the electric corrosion of concrete specimens with a single steel bar at the corner, the current flows from the steel bar to the stainless steel wire on both sides, and the current is superimposed on the surface of the steel bar. As shown in Figure 6, when *s* < *d*, the fitting results show a bimodal shape; when *s* ≥ *d*, the fitting results show a single peak shape. The results in Figure 6 show that the surface current density of a single corner reinforcement can be fitted by the superposition of two Gaussian functions, and the fitting effect is good. The symmetric axes of the two Gaussian functions are *θ* = 0 and *θ* = *π*/2, respectively; so, it is assumed that the distribution of current density is

(14) $$i\left(\theta \right)=y_{1}+T_{m1}\cdot e^{{-\left(\frac{\theta }{\sqrt{2}\cdot c_{1}}\right)}^{2}}+T_{m2}\cdot e^{{-(\frac{\theta -\frac{\pi }{2}}{\sqrt{2}\cdot c_{2}})}^{2}}$$

In the formula, *y*, *T_m_*_1_, *T_m_*_2_, *c*_1_ and *c*_2_ are important coefficients reflecting the current density distribution. The fitted coefficients for the current density distribution of the reinforcement of the 25 specimens are listed in Table 3. As shown in Table 3, compared with the surface current density distribution of a single central steel bar, it is found that *y*_1_ is approximately twice that of *y*; *T_m_*_1_ and *T_m_*_2_ are approximately *T*_m_; and *c*_1_ and *c*_2_ are approximately *c*. Therefore, the electric conduction process of a single corner reinforcement can be regarded as the superposition of the two single middle reinforcements, and the distribution of the surface current density can be expressed as

(15) $$i\left(\theta \right)=2y+T_{m}\cdot \left[e^{{-\left(\frac{\theta }{\sqrt{2}\cdot c}\right)}^{2}}+e^{{-\left(\frac{\theta -\pi /2}{\sqrt{2}\cdot c}\right)}^{2}}\right]$$

#### 3.2.3. Single Row of Multiple Reinforcements

**Figure 7:**
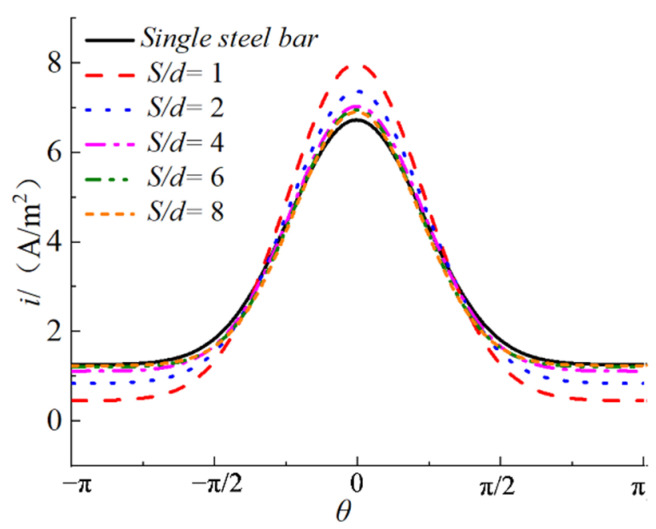
Variation of current density distribution on steel bar surface with *S/d*.

Figure 7 shows the typical results of the variation of the current density distribution on the steel surface with *S*/*d* when multiple reinforcements are electrified and corroded (*d* = 8 mm). It can be seen from Figure 7 that with the increase of *S*/*d*, the maximum current density on the surface of the steel bar gradually decreases, and the minimum current density gradually increases. The distribution of the current density is more dispersed and closer to the current density distribution for a single steel bar. In order to describe the difference of the current density distribution between the multiple bar and the single bar, *Y*, *T*, and *C* are defined as the change rates of the uniform coefficient, the non-uniform coefficient, and the expansion coefficient:

(16) $$Y=\frac{y_{1}-y}{y}\times 100\%$$

(17) $$T=\frac{T_{m1}-T_{m}}{T_{m}}\times 100\%$$

(18) $$C=\frac{c_{1}-c}{c}\times 100\%$$

**Figure 8:**
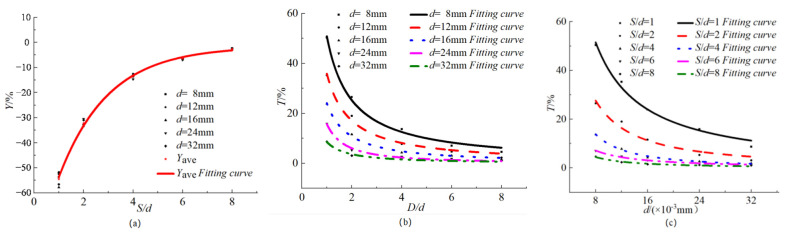
Variation of *Y*, *T*, *C* with *S/d* and *d:* (**a**) variation of *Y* with *S/d*; (**b**) variation of *T* with *S/d*; (**c**) variation of *T* with *d*.

Table 4 shows the three coefficients and their change rates according to the fitting results of the current density distribution of the specimens. It can be seen from Table 4 that the absolute value of the change rate of the expansion coefficient *C* is less than 5%; so, the influence of the reinforcement spacing on the expansion coefficient can be ignored. The relationship of *S/d*, *d*, *Y*, and *T* is obtained, as shown in Figure 8. It can be seen from Figure 8a that *Y* is always negative, indicating that the presence of steel bars on both sides in the process of the power supply will enhance the non-uniformity of the current density distribution of the steel bars in the middle. With the increase of *S/d*, *Y* gradually increases to close to 0, indicating that the larger the net distance of reinforcement, the smaller the degree of non-uniformity enhancement. In addition, the data of the same *S/d* are very close, indicating that *d* has little effect on *Y*, which can be ignored. Therefore, we take the average value of the five data under the same *S/d* as the final value, denoted as *Y*_ave_. It is found that the relationship between *Y*_ave_ and *S/d* conforms to the exponential function. The curve in Figure 8a was fitted by Equation (19), where *S* and *d* are in m.

(19) $$Y\left(\frac{S}{d}\right)=-1.6-86.1e^{-\frac{S/d}{2}},\frac{S}{d}\in \left[1,8\right]$$

The relationship of *T*, *S*/*d*, and *s* is obtained by fitting the results in Table 4, as shown in Equation (20), in which the unit of *S* and *d* is m.

(20) $$T\left(\frac{S}{d},d\right)=0.073-0.14\frac{S}{d}-29.82d+0.01{\left(\frac{S}{d}\right)}^{2}+368.06d^{2}+1.97S,S/d\in \left[1,8\right]$$

The three change rates are all closer to 0 with the increase of *S*/*d*. When *S*/*d* is 8, the absolute values of the three change rates are less than 5%, which can be ignored. The existence of the steel bars on both sides in the process of power transmission will make the current density distribution of the middle steel bars more uneven. However, with the increase in the net distance of the steel bars, this effect becomes smaller. When the net distance of the steel bars is greater than or equal to eight times the diameter, this effect can be ignored. Based on the model of the current density distribution on the surface of the steel bar under the condition of the electrified single steel bar (Equation (13)), combined with Equations (16) to (20), the model of the current density distribution on the surface of the steel bar under the condition of the electrified multiple steel bar is obtained when the net distance between the stainless steel wire and the steel bar is 8 mm:

(21) $$i\left(\theta ,d,\frac{S}{d}\right)=\left[Y\left(\frac{S}{d}\right)+1\right]\cdot y\left(d\right)+\left[T\left(\frac{S}{d},d\right)+1\right]\cdot T_{m}\left(d\right)\cdot e^{{-\left(\frac{\theta }{\sqrt{2}\cdot c\left(d\right)}\right)}^{2}}$$

### 3.3. Thickness Distribution of Rust Layer in Circumferential Direction

Combining Equations (4), (13), (15), and (21) leads to the rust thickness distribution model in the circumferential direction of the ribs. When a single steel bar is corroded, considering the influence of the position and diameter of the steel bar and the net distance between the steel bar and the stainless steel wire, the thickness distribution models of the rust layer, when the steel bar is located in the middle and corner of the specimen, are obtained as in Equation (22) and Equation (23), respectively.

(22) $$T_{r,Z}\left(\theta ,d,s,\rho \right)=\frac{n\cdot d\cdot \rho }{4i_{\mathrm{ave}}}\cdot \left[y\left(d,s\right)+T_{m}\left(d,s\right)\cdot e^{{-\left(\frac{\theta }{\sqrt{2}\cdot c\left(d,s\right)}\right)}^{2}}\right]$$

(23) $$T_{r,D}\left(\theta ,d,s,\rho \right)=\frac{n\cdot d\cdot \rho }{4i_{\mathrm{ave}}}\cdot \left\{2y\left(d,s\right)+T_{m}\left(d,s\right)\cdot \left[e^{{-\left(\frac{\theta }{\sqrt{2}\cdot c\left(d,s\right)}\right)}^{2}}+e^{{-\left(\frac{\theta -\pi /2}{\sqrt{2}\cdot c\left(d,s\right)}\right)}^{2}}\right]\right\}$$

where *n* is the expansion coefficient of the corrosion product (*n* = 2); *d* is the initial diameter of the steel bar (m); *ρ* is the corrosion rate of the steel bar; *i*_ave_ is the average current density of the steel bar surface (A/m^2^); and *y*(*d*,*s*), *T_m_*(*d*,*s*), and *c*(*d*,*s*) are defined in Equation (14).

The net distance between the steel bar and the stainless steel wire is determined as 8 mm when the multiple steel bars are corroded. Considering the influence of the steel bar diameter and the net distance between the steel bar and the stainless steel wire, the model formula of the rust layer thickness distribution is obtained.

(24) $$T_{r}\left(\theta ,d,\frac{S}{d},\rho \right)=\frac{n\cdot d\cdot \rho }{4i_{\mathrm{ave}}}\cdot \left[\left[Y\left(\frac{S}{d}\right)+1\right]\cdot y\left(d\right)+\left[T\left(\frac{S}{d},d\right)+1\right]\cdot T_{m}\left(d\right)\cdot e^{{-\left(\frac{\theta }{\sqrt{2}\cdot c\left(d\right)}\right)}^{2}}\right]$$

where *Y*(*S/d*) and *T*(*S/d,d*) are calculated by Equations (19) and (20). *y*(*d*), *T_m_*(*d*), *c*(*d*) are obtained by substituting *s* = 8 mm into *y*(*d,s*), *T_m_*(*d,s*), and *c*(*d,s*).

**Figure 9:**
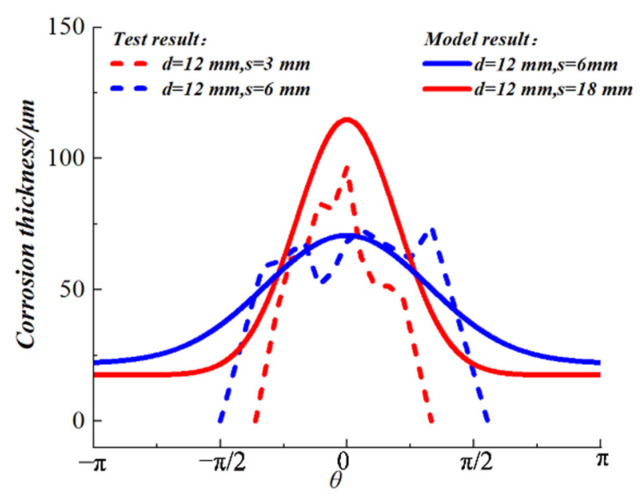
Comparison of the thickness distribution of the rust layer obtained by test and model.

The experimental result of Fu et al. [17], based on the non-uniform corrosion method, was used to verify the thickness distribution model of the rust layer proposed in this study. In their test, ordinary Portland cement mortar was used and the embedded steels (the chemical compositions of the steel and cement are shown in Table 5 and Table 6, respectively) were hot-rolled plain bars with a diameter of 6 mm. The non-uniform rust distribution was produced based on the impressed current method. The rust thickness distribution was detected by X-ray microtomography. The comparison between the experimental results and the model results is shown in Figure 9. The experimental results are in good agreement with the model fitting results, and the model results are slightly larger than the experimental results. This is because the current will be lost in the process of concrete transmission, resulting in the actual corrosion rate being slightly less than the theoretical value.

## 4. Corrosion Expansion Cracking for Non-Uniformly Corroded Reinforcement

### 4.1. Simulation Method

**Figure 10:**
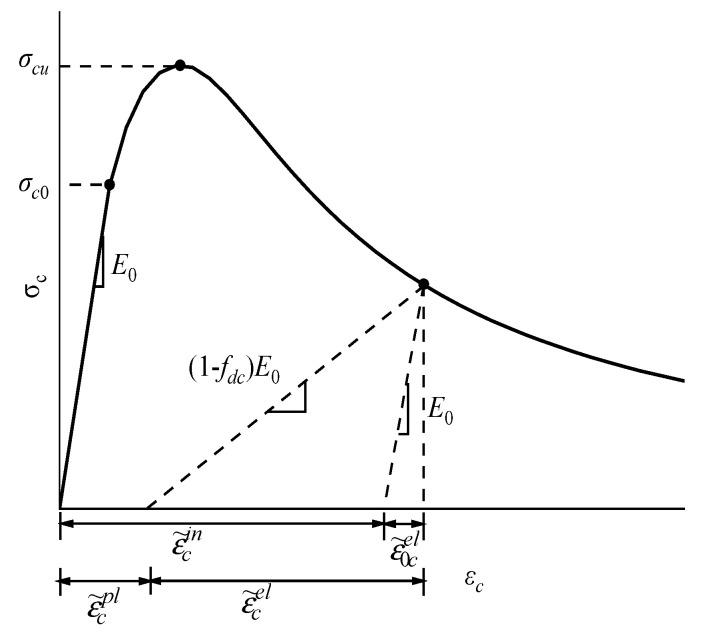
Stress–strain curve of concrete under uniaxial compression based on plastic damage model.

When the plastic damage model is adopted in ABAQUS [13,22], the stress–inelastic strain curve and the inelastic strain–damage factor curve under axial tension and compression need to be inputted. Figure 10 shows the stress–strain curve of the concrete under uniaxial compression defined by the plastic damage model. In the elastic stage, the stress increases linearly with the strain and enters the strengthening stage after reaching the yield stress *σ_c_*_0_. The stress continues to increase and enters the softening stage after reaching the ultimate stress *σ_cu_*. The stress is unloaded according to the degradation stiffness of (1 − *f*_d__c_)*E*_0_, where there is the compression damage factor. From Figure 10, the equivalent plastic strain of the concrete under axial compression can be expressed as [22]:

(25) $${\tilde{\varepsilon }}_{c}^{pl}=\varepsilon_{c}^{in}-\frac{f_{d}{}_{c}}{1-f_{d}{}_{c}}\cdot \frac{\sigma_{c}}{E_{0}}$$

where *σ_c_* is the tensile stress of the concrete; and *ε_c_^in^* is an inelastic strain under axial compression, which can be calculated by Formula (26).

(26) $${\tilde{\varepsilon }}_{c}^{in}=\varepsilon_{c}-\frac{\sigma_{c}}{E_{0}}$$

where *ε_c_* is the compressive strain of the concrete.

The stress–strain relationship of the concrete under uniaxial tension and compression is, respectively, Formulas (27) and (28):

(27) $$\sigma_{t}=\left(1-f_{\mathrm{dt}}^{}\right)\cdot E_{c}\cdot \varepsilon_{t}$$

(28) $$\sigma_{c}=\left(1-f_{\mathrm{dc}}\right)\cdot E_{c}\cdot \varepsilon_{c}$$

where *E_c_* is the elastic modulus of the concrete represented by its strength grade (*f_cu__,__k_*), which can be calculated according to Formula (29), and the unit is N/mm^2^; *d_t_* and *d_c_* are the tensile damage factor and the compressive damage factor, respectively, which can be calculated by Formulas (30) and (31) [23].

(29) $$E_{c}=\frac{10^{5}}{2.2+\frac{34.7}{f_{cu,k}}}$$

(30) $$f_{d}{}_{t}=\left\{ \begin{array}{l} 1-\rho_{t}\cdot \left(1.2-0.2x\right),x\leq 1\\ 1-\frac{\rho_{t}}{1-\alpha_{t}\cdot {\left(x-1\right)}^{1.7}+x},x>1 \end{array}\right.$$

(31) $$f_{d}{}_{c}=\left\{ \begin{array}{l} 1-\frac{\rho_{c}\cdot n}{n-1+x^{n}},x\leq 1\\ 1-\frac{\rho_{c}}{\alpha_{c}\cdot {\left(x-1\right)}^{2}+x},x>1 \end{array}\right.$$

where *α_t_*, *α_c_* can be selected in specification [24]; *x*, *ρ_t_*, *ρ_c_*, and *n* can be calculated as follows:

(32) $$x=\frac{\varepsilon }{\varepsilon_{t/c,r}}$$

(33) $$\rho_{t/c}=\frac{f_{t/c,r}}{E_{t/c}\cdot \varepsilon_{t/c,r}}$$

(34) $$n=\frac{E_{c}\cdot \varepsilon_{c,r}}{E_{c}\cdot \varepsilon_{c,r}-f_{c,r}}$$

The damage factors of the concrete under tension and compression can be calculated by the stress–strain curve according to the principle of energy equivalence. As the input in ABAQUS is the softening stage of the stress–strain curve, the equivalent plastic strain must be greater than or equal to the inelastic strain. According to Formula (25), *D_t_* and *D_c_* must be greater than or equal to 0. Therefore, the expressions of *D_t_* and *D_c_* are given by

(35) $$D_{t}=1-\sqrt{\left(1-f_{d}{}_{t}\right)\frac{E_{t}}{E_{0}}},D_{t}\geq 0$$

(36) $$D_{c}=1-\sqrt{\left(1-f_{d}{}_{c}\right)\frac{E_{c}}{E_{0}}},D_{c}\geq 0$$

where *E*_0_ is the elastic modulus of the concrete in the elastic stage taken in ABAQUS, which can be calculated by Formula (37), according to the specification [23].

(37) $$E_{0}=\frac{f_{t,r}}{\varepsilon_{t,r}}$$

According to Formulas (25)–(37), the stress–inelastic strain curve and the inelastic strain–damage factor curve of the axial tension and compression can be obtained. In the process of finite element analysis, the equivalent plastic tensile strain is used to describe the development of cracks after concrete cracking. Concrete cracking is defined to occur when the equivalent plastic tensile strain is greater than 0. The relevant parameters of concrete C30 are shown in Table 6. In Table 7, *Ψ* is the expansion angle, *ε* is the eccentricity of the potential function, *σ_b_*_0_/*σ_c_*_0_ is the ratio of the biaxial compression and the uniaxial compression of the concrete, *K* is the ratio of the second stress constant on the tensile meridian to the compressive meridian, *μ* is the viscosity coefficient, *E_c_* is the elastic modulus of the concrete, and *v* is Poisson’s ratio.

The main influencing factors of the single steel bar cracking pattern are the steel bar position (middle position or corner position), the steel bar diameter, the concrete cover thickness, and the steel bar–stainless steel wire net distance. The diameter of reinforcement, the thickness of the concrete cover and the net distance between the reinforcement and the stainless steel wire are shown in Table 8. The main influencing factors of the multiple steel bar cracking patterns are the steel bar diameter, the steel bar spacing, and the concrete cover thickness. The net distance between the reinforcement and the stainless steel wire is 8 mm. The diameter of the reinforcement, the net distance between the reinforcements, the thickness of the concrete protective layer, and the net distance between the reinforcement and the stainless steel wire are shown in Table 9. The rust expansion cracking of the reinforced concrete can be treated according to the plane strain problem. The finite element analysis model adopts the two-dimensional analysis model, and the equal diameter cavity is used to replace the position of the reinforcement. The concrete section size of the single steel bar is 150 × 150 mm, and the concrete section size of the multiple steel bars is 300 × 300 mm. As the stress and strain around the reinforcement are relatively concentrated, the mesh around the reinforcement is appropriately encrypted. The analysis model uses the method of applying radial displacement to simulate the non-uniform corrosion expansion of the reinforcement. In order to facilitate the calculation, the compression of the corrosion products and the effect of filling the concrete voids are not considered.

### 4.2. Influence of Geometric Parameters of Steel Electrode

As mentioned in Section 3, the distribution of the current density and the corresponding corrosion thickness depend on the geometric parameters, including the diameter of the steel bar and the distance between the steel bar and the stainless steel wire. By controlling the value of the geometric parameters, the distribution of the rust layer thickness can be designed, thus affecting the cracking behavior of the concrete. In this paper, the effect of the geometric parameters of a steel electrode on the cracking behavior is discussed from two aspects: the horizontal crack angle (α) and the critical corrosion degree (*η*_s_, *c*_r_) of the concrete surface cracking.

**Figure 11:**
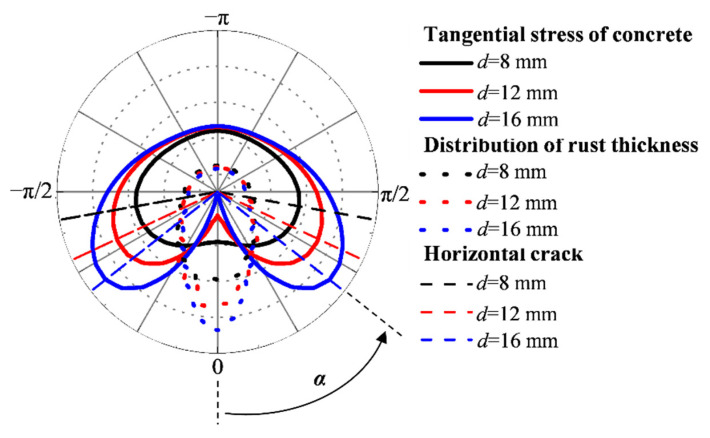
Rust layer distribution and crack morphology of steel bars with different diameters.

**Figure 12:**
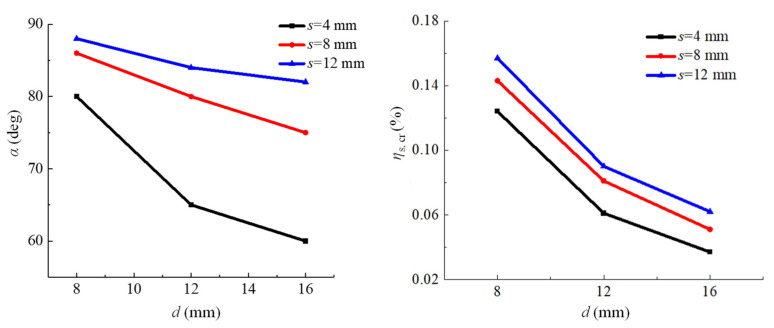
Relationship between horizontal crack angle (*α*) and critical corrosion degree (*η*s, *c*r) and steel bar diameter (*d*).

**Figure 13:**
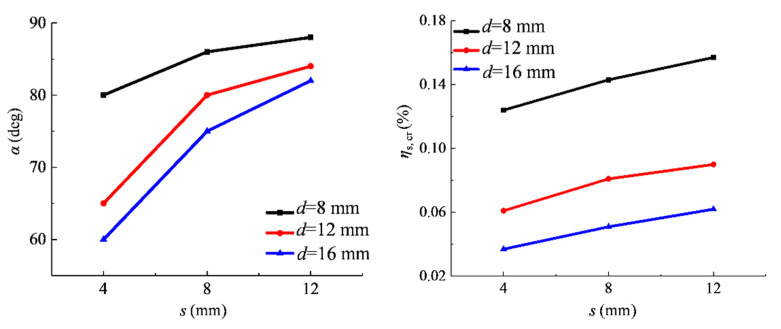
Relationship between angle (*α*) and critical corrosion degree (*η*s, *c*r) of horizontal cracks and distance (*s*) between steel bars and stainless steel wires.

In the case of the other parameters being unchanged, with the increase of the steel bar diameter, the uneven coefficient of the current density increases, while the uniformity coefficient and the distribution coefficient of the current density decrease. In this regard, the larger the diameter, the higher the unevenness of the current density, and the corresponding corrosion thickness around the steel bar is also larger. As shown in Figure 11, with the increase in diameter, the maximum value of the corrosion thickness gradually increases, resulting in a greater tangential stress of concrete and a slight change in the direction of the maximum tangential stress. Therefore, with the increase of the steel diameter, the horizontal crack angle and the critical corrosion degree related to the external surface cracking decrease, as shown in Figure 12. Similarly, when the distance between the steel and stainless steel wire increases, the current density non-uniformity level and the corrosion thickness around the steel bar decrease. It can be seen from Figure 13 that with the increase in distance, the horizontal crack angle and the critical corrosion degree increase.

### 4.3. Effect of Steel Bar Position

**Figure 14:**
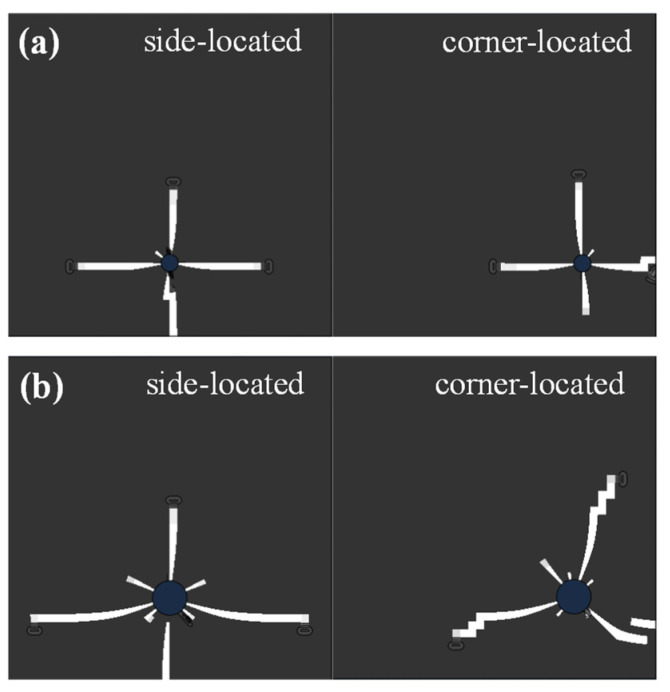
Crack morphology of side and corner of steel bar: (**a**) *s* = 12 mm, *d* = 8 mm, *c* = 30 mm; (**b**) *s* = 8 mm, *d* = 16 mm, *c* = 30 mm.

Figure 14 shows the crack distribution of the steel bars at different locations. For side reinforcement, the cracks are mainly distributed in the horizontal and vertical directions in this study. For the steel at the corner, as shown in Figure 14, the crack pattern is changed by changing the relationship between *s* and *d*. In the case of *s* > *d* (Figure 14b), the number of primary cracks changes, and the cracks no longer propagate horizontally and vertically.

**Figure 15:**
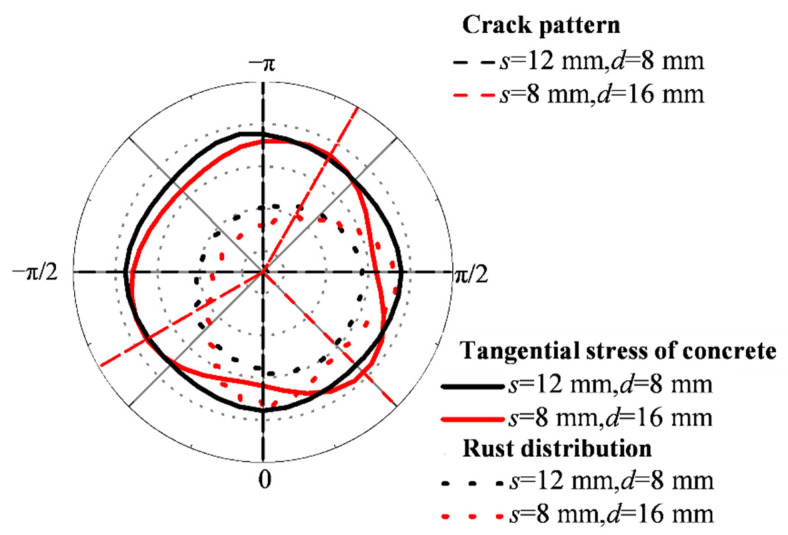
Distribution of rust layer and crack pattern of corner-located steel under non-uniform accelerated corrosion.

As shown in Figure 15, the steel bars on the side subjected to the non-uniform accelerated corrosion show a single corrosion peak, that is, the maximum thickness of the rust layer. The change of the relationship between *s* and *d* leads to different values of the uniform coefficient, the non-uniform coefficient, and the diffusion coefficient of the rust layer. However, in this case, the number of corrosion peaks remains one. For the steel at the corner, the number of corrosion peaks is related to the relationship between *s* and *d*. In the case of *s* > *d* (i.e., *s* = 12 mm, *d* = 8 mm), a corrosion peak appears near *θ*. Therefore, the concrete on both sides near *θ* bears the maximum tangential stress, resulting in mainly horizontal and vertical cracks. For *s* < *d* (i.e., *s* is 8 mm, *d* is 16 mm), two corrosion peaks appear in the two corrosion directions of *θ* = 0 and *θ* = π/2. Due to the combined action of these two corrosion peaks, the concrete around *θ* = π/4 suffered the maximum tangential stress. Therefore, the crack pattern of the corner steel changes according to the relationship between *s* and *d*.

### 4.4. Effect of Reinforcement Spacing

**Figure 16:**
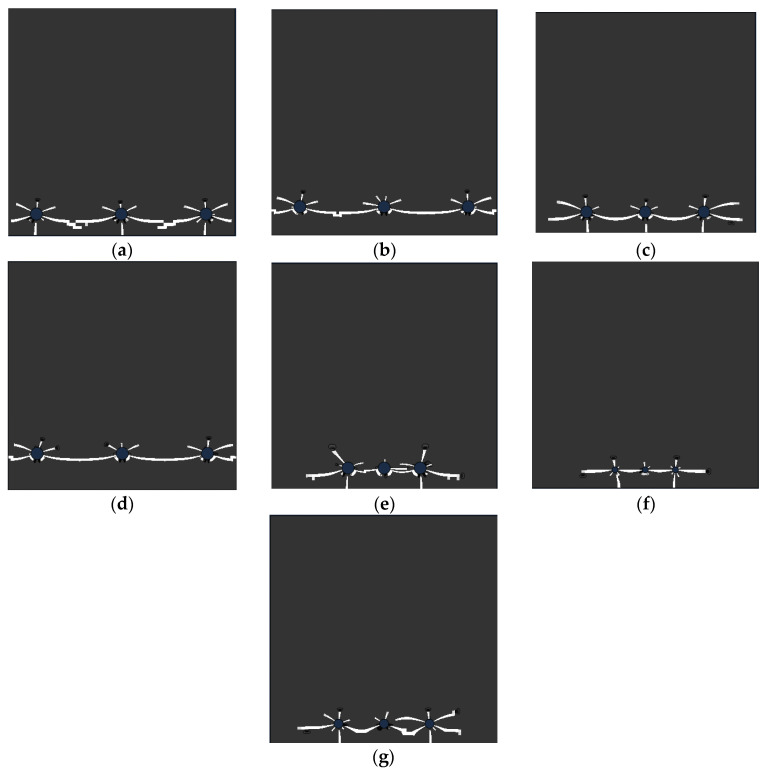
Typical crack distribution of concrete reinforced by multiple steels: (**a**) *d* = 16 mm, *S* = 6*d*, *c* = 20 mm; (**b**) *d* = 16 mm, *S* = 6*d*, *c* = 30 mm; (**c**) *d* = 16 mm, *S* = 6*d*, *c* = 40 mm; (**d**) *d* = 16 mm, *S* = 4*d*, *c* = 20 mm; (**e**) *d* = 16 mm, *S* = 2*d*, *c* = 20 mm; (**f**) *d* = 8 mm, *S* = 4*d*, *c* = 20 mm; (**g**) *d* = 12 mm, *S* = 4*d*, *c* = 20 mm.

Considering the non-uniform corrosion of the single row of multiple plain steel bars, the influence of the interaction of the surrounding steel bars on the cracking pattern of the concrete was mainly explored. Therefore, three adjacent reinforcements were selected for simulation to explore the cracking pattern of the concrete around the middle reinforcement. According to the finite element analysis results of the multiple smooth circle reinforced concrete, the crack distribution results of some of the typical concrete structures are listed, as shown in Figure 16. It can be seen that the distribution of cracks in the concrete around the middle reinforcement is divided into two types: (1) horizontal linear distribution, where the failure pattern of the concrete is the overall spalling failure of the protective layer and (2) tree-shaped distribution, where the failure pattern of the concrete is the penetration failure of the protective layer. The results show that: when *S* < 3*c*, the fracture has a horizontal linear distribution; when *S* > 3*c*, the cracks are distributed in a branch shape.

### 4.5. Effect of Steel Morphology

**Figure 17:**
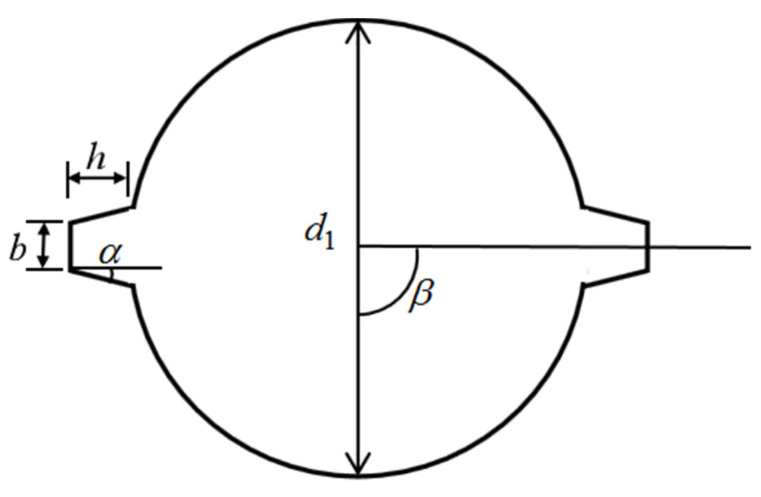
Schematic diagram of ribbed steel bar cross section.

**Figure 18:**
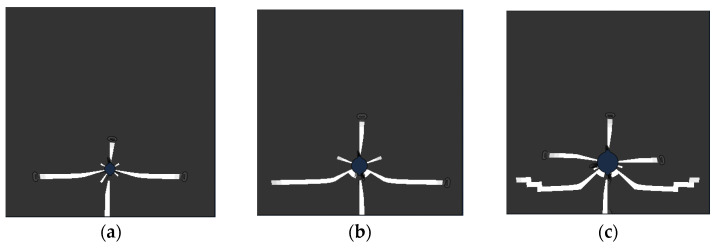

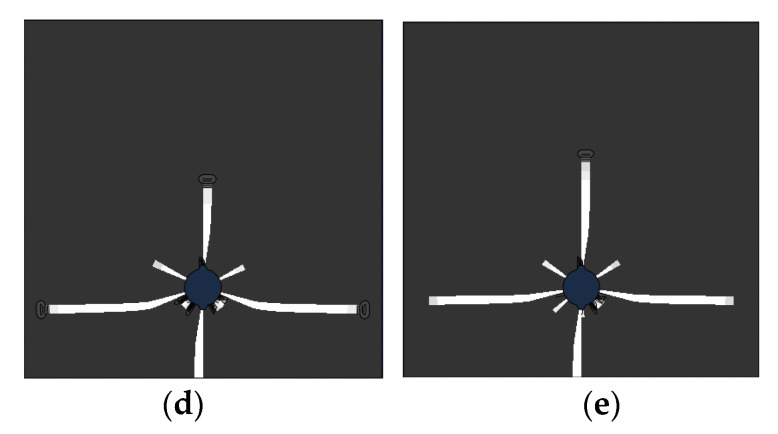
Crack distribution of single central-ribbed reinforced concrete (*β* = 0): (**a**) *s* = 4 mm, *d* = 8 mm; (**b**) *s* = 4 mm, *d* = 12 mm; (**c**) *s* = 4 mm, *d* = 16 mm; (**d**) *s* = 8 mm, *d* = 16 mm; (**e**) *s* = 12 mm, *d* = 16 mm.

**Figure 19:**
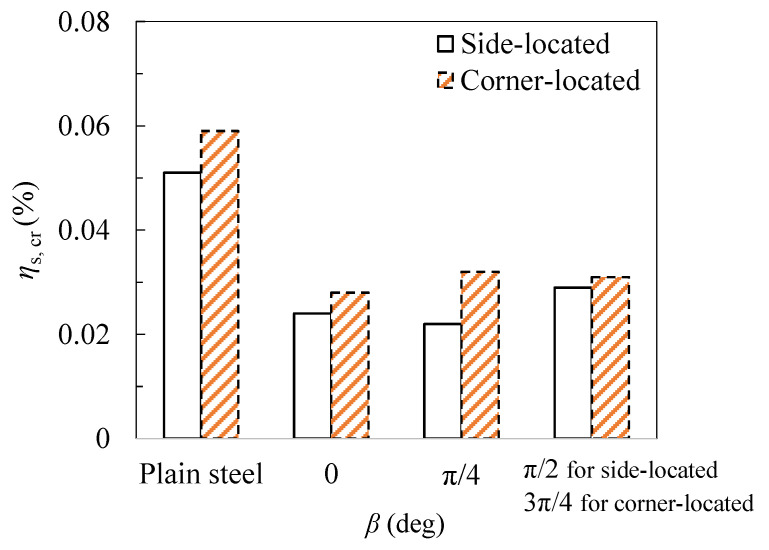
Critical corrosion degree under different rib angles (*s* = 8 mm, *d* = 16 mm, *c* = 30 mm).

In order to study the influence of the steel shape on the corrosion thickness distribution and cracking behavior, the ribbed steel bars with a geometric shape, as shown in Figure 17, were fabricated. Table 10 lists the values of the different rib angles. Three angles (β = 0, π/4, π/2) were studied for the side steel, simulating one-dimensional chloride corrosion. Due to the symmetrical position of the angular steel in the two-dimensional chloride ion corrosion, the angles of 0, π/4, and 3π/4 were studied. Figure 18 shows the crack modes of the steel with different rib angles. Stress concentration occurs on the ribs, resulting in cracks. Therefore, the crack pattern of the concrete depends on the angle of the ribs. On the other hand, due to the existence of the ribs, the tangential stress of the concrete increases and the critical corrosion degree decreases, as shown in Figure 19. However, slight changes in the critical corrosion degree were observed by changing the rib angle.

## 5. Conclusions

In this study, a model for calculating the thickness distribution of the rust layer in the non-uniform electrochemical accelerated corrosion was established. The experimental results of the non-uniform accelerated corrosion method verified the thickness distribution model of the rust layer. Based on the simulated results, the following conclusions can be drawn.

- The distribution of the surface current density of the steel bar under non-uniform corrosion conforms to the Gaussian function with the determination coefficient of 0.99. The non-uniformity of the current density distribution increases by increasing the diameter of the steel bars or reducing the net distance between the steel bars and the stainless steel bars.
- For the multiple steel scenario, the distribution of the current density in the middle of the steel bar is more uneven due to the existence of both sides of the steel bar in the process of multiple steel bars. However, when the ratio of the net distance of the steel bar to the diameter *S*/*d* was greater than 8, the change rates were all smaller than 5%, indicating that this effect can be ignored.
- As the position of the steel bar differed, the crack pattern of the concrete changed, depending on the number of corrosion peaks (i.e., the maximum thickness of the rust layer). In terms of the corner-located steel, the number of corrosion peaks varied in the cases of different geometrical parameters (i.e., the diameter of the steel bar and the distance between the steel bar and the stainless steel wire).
- The critical corrosion degree for the ribbed steel bar with an angle of π/4 at the side can be 56% smaller than that for the plain steel bar. The tangential stress of the concrete increases due to the existence of ribs, which reduce the critical corrosion degree. The critical corrosion degrees of the side-located steel bars can be about 35% smaller than those of the corner-located steel bars.
- When considering the non-uniform corrosion of a single row of multiple plain bars, the distribution types of the concrete-cracking cracks around the middle bars are divided into two types. When *S* < 3*c*, the fracture has a horizontal linear distribution; when *S* > 3*c*, the crack has a branch-shaped distribution.

The rust layer thickness distribution and the corrosion-induced cracking patterns were studied based on the FEM simulation. However, the simulation was based on the experimental setup for accelerated corrosion methods, and its relations to the corrosion under natural conditions should be further investigated. Furthermore, the time-dependent corrosion rate needs to be considered in the future.

## Figures and Tables

**Table 1:** Finite element simulation parameters of galvanic corrosion of steel bars.

Parameter	Symbol	Value	Unit
External current density	*i*	3	A/m^2^
Anode equilibrium potential	*E* ^0^ *_a_*	−0.618	V
Cathode equilibrium potential	*E* ^0^ *_c_*	−0.414	V
Anode exchange current density	*I* ^0^ *_a_*	7.1	A/m^2^
Cathodic exchange current density	*I* ^0^ *_c_*	110	A/m^2^
Anode charge transfer coefficient	*α*	0.5	1
Cathode charge transfer coefficient	*γ*	0.5	1
Temperature	*T*	298	K

**Table 2:** Fitting values of parameters of Gauss model for side laying reinforcement.

*s* (×10^−3^ m)	*d* (×10^−3^ m)	*y*	*T_m_*	*c*
4	8	1.277	6.457	0.655
	12	1.140	8.822	0.511
	16	1.039	11.656	0.409
	24	0.912	16.657	0.298
	32	0.860	21.530	0.242
8	8	1.450	4.269	0.873
	12	1.281	5.483	0.744
	16	1.145	6.909	0.636
	24	0.951	9.639	0.498
	32	0.881	12.552	0.411
12	8	1.623	3.330	0.986
	12	1.399	4.485	0.843
	16	1.228	5.343	0.768
	24	1.015	7.216	0.645
	32	0.916	9.322	0.551
16	8	1.756	2.887	1.132
	12	1.486	3.606	0.979
	16	1.305	4.549	0.863
	24	1.083	6.257	0.723
	32	0.937	7.945	0.640
20	8	1.852	2.482	1.206
	12	1.547	3.200	1.028
	16	1.336	4.0307	0.935
	24	1.117	5.552	0.785
	32	0.957	7.177	0.700

**Table 3:** Fitting of current density distribution on the surface of single corner bar.

*s* (×10^−3^ m)	*d* (×10^−3^ m)	Fitted Coefficients				
		*y*_1_	*T_m_*_1_	*T_m_*_2_	*c*_1_	*c*_2_
4	8	2.517	6.641	6.587	0.655	0.688
	12	2.238	8.851	8.739	0.535	0.498
	16	1.834	12.340	12.218	0.400	0.422
	24	1.800	16.770	16.715	0.282	0.293
	32	1.705	21.543	21.466	0.236	0.240
8	8	3.012	4.261	4.281	0.805	0.832
	12	2.659	5.548	5.671	0.746	0.751
	16	2.299	7.203	7.151	0.606	0.620
	24	1.822	9.669	9.632	0.494	0.485
	32	1.778	12.274	12.257	0.411	0.417
12	8	3.279	3.335	3.296	0.989	0.920
	12	2.873	4.496	4.584	0.824	0.836
	16	2.524	5.486	5.351	0.782	0.784
	24	2.046	7.259	7.283	0.647	0.631
	32	1.772	9.271	9.455	0.530	0.519
16	8	3.465	2.868	2.841	1.007	1.010
	12	3.030	3.745	3.665	0.979	0.907
	16	2.691	4.602	4.584	0.858	0.866
	24	2.189	6.402	6.311	0.698	0.710
	32	1.800	8.142	8.151	0.622	0.639
20	8	3.885	2.461	2.416	1.131	1.103
	12	3.056	3.187	3.210	1.084	1.093
	16	2.688	4.137	4.147	0.947	0.940
	24	2.263	5.588	5.467	0.781	0.771
	32	1.902	7.126	7.123	0.691	0.696

**Table 4:** Fitting the distribution of current density on the surface of steel bars in the case of multiple steel bars.

*d* (×10^−3^ m)	*S*/*d*	Fitted Coefficients			Change Rate		
		*y*_1_	*T_m_*_1_	*c*_1_	*Y* (%)	*T* (%)	*C* (%)
8	1	0.671	6.419	0.886	−53.724	50.363	1.489
	2	0.966	5.399	0.872	−33.379	26.470	−0.115
	4	1.267	4.855	0.848	−12.621	13.727	−2.864
	6	1.351	4.566	0.834	−6.828	6.957	−4.467
	8	1.411	4.467	0.832	−2.690	4.638	−4.696
12	1	0.556	7.416	0.752	−56.596	35.254	1.075
	2	0.853	6.523	0.742	−33.411	18.968	−0.269
	4	1.107	5.913	0.724	−13.583	7.842	−2.688
	6	1.203	5.741	0.715	−6.089	4.705	−3.898
	8	1.251	5.607	0.708	−2.342	2.262	−4.839
16	1	0.483	8.559	0.654	−57.817	23.882	2.830
	2	0.773	7.708	0.653	−32.489	11.565	2.673
	4	0.992	7.206	0.621	−13.362	4.299	−2.358
	6	1.075	7.124	0.606	−6.114	3.112	−4.717
	8	1.114	7.012	0.605	−2.707	1.491	−4.874
24	1	0.451	11.175	0.522	−52.576	15.935	4.819
	2	0.661	10.153	0.507	−30.494	5.333	1.807
	4	0.811	9.901	0.489	−14.721	2.718	−1.807
	6	0.884	9.819	0.482	−7.045	1.867	−3.213
	8	0.928	9.756	0.475	−2.419	1.214	−4.618
32	1	0.425	13.648	0.430	−51.759	8.732	4.623
	2	0.608	12.941	0.419	−30.988	3.099	1.946
	4	0.766	12.789	0.403	−13.053	1.888	−1.946
	6	0.824	12.686	0.400	−6.470	1.068	−2.676
	8	0.860	12.682	0.400	−2.384	1.036	−2.676

**Table 5:** Chemical composition of steel (mass %).

	C	Si	Mn	P	S
Steel	0.25	0.55	1.5	0.045	0.05

**Table 6:** Chemical composition of cement (mass %).

Composition	Content
CaO	57.00
SiO_2_	23.41
Al_2_O_3_	5.50
SO_3_	2.56
Fe_2_O_3_	3.60
MgO	2.75
Loss on ignition	5.14

**Table 7:** Parameters of concrete materials.

*Ψ*	*ε*	*σ_b_*_0_/*σ_c_*_0_	*K*	*µ*	*E_c_* (MPa)	*v*	*F_t,r_*	*E_t,r_*
30°	0.1	1.16	2/3	0.0005	30,000	0.2	2.01	9.524 × 10^−5^

**Table 8:** Influencing parameters of corrosion-cracking model of single plain reinforced concrete.

Position	*d/*mm	*c*/mm	*s*/mm
Central	6	20	4
	12	30	8
Corner	16	40	12

**Table 9:** Affecting parameters of corrosion cracking for multiple plain reinforced concrete.

*d/*mm	*c*/mm	*S*/mm
8	20	2
12	30	4
16	40	6

**Table 10:** Details of ribbed steel bar.

Position of Steel	*d* (mm)	*s* (mm)	*β* (deg)
Side-located	16	8	0
	16	8	π/4
	16	8	π/2
Corner-located	16	8	0
	16	8	π/4
	16	8	3π/4

## Data Availability

The data presented in this study are available on request from the corresponding author. The data are not publicly available, due to privacy restrictions.

## Nomenclature

*c*	the width of Gaussian curve
*d*	the initial diameter of reinforcement (m)
*d_a_*	the diameters of anode (m)
*d_c_*	the diameters of cathode (m)
*E_a/c_*	polarization potential (V)
*E* ^0^ *_a/c_*	equilibrium potential (V)
*E_c_*	the elastic modulus of concrete (V)
*F*	Faraday constant (C/mol)
*f_cu,k_*	concrete strength grade (N/mm^2^)
*f_dt_*	tensile damage factor
*f_dc_*	compressive damage factor
*I*	constant current (A)
*i_a_*	the corrosion current densities of anode (A/m^2^)
*i*_ave_	the average current density on the steel surface (A/m^2^)
*i_c_*	the corrosion current densities of cathode (A/m^2^)
*i* ^0^ *_a_*	the exchange current densities of anode (A/m^2^)
*i* ^0^ *_c_*	the exchange current densities of cathode (A/m^2^)
*K*	the ratio of the second stress constant on the tensile meridian to the compressive meridian
*l*	the length of the electrified area of the steel bar (m)
*M*_Fe_	molar mass of iron (g/mol)
*m*	the combined price
*n*	expansion coefficient of rust products
*R*	a gas constant (J/(mol·K))
*S*	reinforcement spacing (mm)
*s*	the net distance between the two electrodes (mm)
*T*	the thermodynamic temperature (K)
*T_m_*	the inhomogeneity of current density
*V*	volume of corrosion products (m^3^)
*t*	test time (s)
*y*	the minimum current density (A/m^2^)
*Z*_Fe_	the combined valence of iron
*α*	the charge transfer coefficients of anode
*β*	rib angles
*γ*	the charge transfer coefficients of cathode
*γ_s_*	steel density (g/m^3^)
*ε*	the eccentricity of the potential function
*ε_c_^in^*	an inelastic strain under axial compression
*σ*_*b*0_/*σ*_*c*0_	the ratio of biaxial compression and uniaxial compression of concrete
*ε_c_*	compressive strain of concrete
*v*	Poisson’s ratio
*ρ*	the corrosion rate of the steel bar
*σ_c_*	tensile stress of concrete (N/mm^2^)
*η_a/c_*	overpotential (V)
*μ*	the viscosity coefficient
*Ψ*	the expansion angle
